# Association Between Finger-to-Nose Kinematics and Upper Extremity Motor Function in Subacute Stroke: A Principal Component Analysis

**DOI:** 10.3389/fbioe.2021.660015

**Published:** 2021-04-12

**Authors:** Ze-Jian Chen, Chang He, Nan Xia, Ming-Hui Gu, Yang-An Li, Cai-Hua Xiong, Jiang Xu, Xiao-Lin Huang

**Affiliations:** ^1^Department of Rehabilitation Medicine, Tongji Hospital, Tongji Medical College, Huazhong University of Science and Technology, Wuhan, China; ^2^World Health Organization Cooperative Training and Research Center in Rehabilitation, Wuhan, China; ^3^State Key Lab of Digital Manufacturing Equipment and Technology, Institute of Rehabilitation and Medical Robotics, Huazhong University of Science and Technology, Wuhan, China

**Keywords:** stroke, upper extremity, kinematics, motor function, principal component analysis

## Abstract

**Background:**

Kinematic analysis facilitates interpreting the extent and mechanisms of motor restoration after stroke. This study was aimed to explore the kinematic components of finger-to-nose test obtained from principal component analysis (PCA) and the associations with upper extremity (UE) motor function in subacute stroke survivors.

**Methods:**

Thirty-seven individuals with subacute stroke and twenty healthy adults participated in the study. Six kinematic metrics during finger-to-nose task (FNT) were utilized to perform PCA. Clinical assessments for stroke participants included the Fugl-Meyer Assessment for Upper Extremity (FMA-UE), Action Research Arm Test (ARAT), and Modified Barthel Index (MBI).

**Results:**

Three principal components (PC) accounting for 91.3% variance were included in multivariable regression models. PC1 (48.8%) was dominated by mean velocity, peak velocity, number of movement units (NMU) and normalized integrated jerk (NIJ). PC2 (31.1%) described percentage of time to peak velocity and movement time. PC3 (11.4%) profiled percentage of time to peak velocity. The variance explained by principal component regression in FMA-UE (*R*^2^ = 0.71) were higher than ARAT (*R*^2^ = 0.59) and MBI (*R*^2^ = 0.29) for stroke individuals.

**Conclusion:**

Kinematic components during finger-to-nose test identified by PCA are associated with UE motor function in subacute stroke. PCA reveals the intrinsic association among kinematic metrics, which may add value to UE assessment and future intervention targeted for kinematic components for stroke individuals.

**Clinical Trial Registration:**

Chinese Clinical Trial Registry (http://www.chictr.org.cn/) on 17 October 2019, identifier: ChiCTR1900026656.

## Introduction

Stroke is the leading cause of disability worldwide, and upper extremity (UE) motor impairment is one of the most relevant functions affected in stroke (Langhorne et al., 2009; GBD, 2019). The impairment results in poor motor control and exerts a negative impact on UE functional capacity and activities of daily living (ADL). To optimize UE recovery after stroke, it is essential to select multilevel outcome measure for interpretation of motor recovery and clinical decision-making (Winstein et al., 2016; Villepinte et al., 2020). According to the International Classification of Functioning, Disability and Health (ICF) (WHO, 2001), there have been extensive validated UE scales on body function and activity, among which the Fugl-Meyer Assessment of Upper Extremity (FMA-UE), Action Research Arm Test (ARAT), and Modified Barthel Index (MBI) are commonly utilized in clinical practice (Santisteban et al., 2016). However, these ordinal rating scales may carry the potential for examiner bias and lack sensitivity to quantify small but potentially impacting change over time (Lang et al., 2013).

Kinematic analysis facilitates interpreting the extent and mechanisms of motor restoration, and it has been increasingly applied in neurological research (Balasubramanian et al., 2012). Although kinematic approaches are objective, sensitive and quantitative, their associations with clinical measures have not been fully studied (Schwarz et al., 2019). In previous studies of kinematic metrics, multivariable regression models are often employed to explain clinical outcomes. Due to the prerequisites of statistical models, such approaches were unable to include high collinear but potentially useful variables. In the case of collinearity, kinematic metrics of lower correlation with dependent variables were removed from the models (Alt Murphy et al., 2012; van Dokkum et al., 2014; Hussain et al., 2019). However, variables in the models measured only limited aspect of UE motor function, hardly to explain heterogeneity in clinical presentations and the intrinsic correlations among kinematic variables during motor recovery (Tran et al., 2018; Schwarz et al., 2019).

Principal component analysis (PCA) is a dimensionality reduction technique to retain the most variance of dataset without the need to exclude highly correlated variables (Zhang and Castelló, 2017). Since principal components (PCs) are the linear combinations of original variables, dataset can be represented as several statistically independent PCs (Ringnér, 2008). To our knowledge, kinematics studies using PCA regression models have focused on the distal hand. In a recent study, representative features of manual dexterity were extracted by a PCA-based logistic regression method (Lin et al., 2019). The model had shown to increase performance in identifying the severity of hand dysfunction in stroke participants. In another study of participants with mild stroke, three PCs in combination, including grip force scaling, motor coordination and speed of movement could predict manipulation skills measured by Jebsen Taylor Hand Function Test (Allgöwer and Hermsdörfer, 2017). PCA is also widely implemented in other clinical researches such as identification of patient phenotypes and prognosis prediction, but is rarely used in UE kinematics (Ibrahim et al., 2014; Badhiwala et al., 2018).

The aim of this study was to explore the kinematic components of finger-to-nose task (FNT) obtained from PCA and the associations with upper extremity motor function in subacute stroke survivors. Furthermore, we hypothesized that kinematic metrics reflected movement strategy, smoothness and velocity during the FNT; hence, the models were considered to measure aspects of motor impairment (FMA-UE), and explain more variance than activity assessments (ARAT and MBI).

## Materials and Methods

### Participants

A total of 37 individuals with subacute stroke (28 men, aged 49.78 ± 10.26 years) and 20 healthy adults (12 men, aged 52.62 ± 10.23 years) were recruited in the study. The inclusion criteria for subacute stroke individuals were: (1) Clinical diagnosis of unilateral, first-ever subacute stroke verified by brain imaging (MRI or CT). (2) Aged between 18 and 80 years. (3) Showing motor impairment (FMA-UE < 66). (4) Mini-Mental State Examination score ≥ 22 and compliance with the assessments. (5) No complicating medical history such as visual, cardiac or pulmonary disorders. Exclusion criteria were other musculoskeletal or neurological conditions that affect arm function. Control participants were 18–80 years old, and had no neurological or orthopedic disorders (Johansson et al., 2017). All participants in this study were right-handed as determined by the Edinburgh Handedness Inventory (Verdino and Dingman, 1998). Data were extracted from the cohort of a clinical study in the Department of Rehabilitation Medicine. We followed the Strengthening the Reporting of Observational Studies in Epidemiology (STROBE) checklist for cross-sectional studies (Vandenbroucke et al., 2007).

### Clinical Assessments

Clinical assessments for stroke participants included the FMA-UE, ARAT, and MBI. The FMA-UE is a reliable and validated measure of motor impairment after stroke. The FMA-UE consists of 33 items (scores ranging from 0 to 66) and higher scores indicate less upper limb impairment (See et al., 2013). The ARAT was used to evaluate functional ability and dexterity of the paretic upper limb. It consists of 19 items (scores ranging from 0 to 57) and higher scores indicate greater arm functional capacity (Yozbatiran et al., 2008). The level of independence in basic activities of living was assessed with the translated version of MBI. The MBI consists of 10 items (scores ranging from 0 to 100), and higher scores indicate greater ADL independence (Leung et al., 2007).

### Kinematic Testing Protocol

The kinematic test was accomplished by a portable Inertial Measurement Unit system (IMU, Noraxon USA Inc.). Each IMU sensor contained a coordinate system to measure accelerations and three-dimensional orientations at a sampling frequency of 100 Hz. The IMU system showed excellent reliability, accuracy and precision in quantifying kinematic testing protocol (Öhberg and Bäcklund, 2019; Park and Lee, 2020). According to a rigid upper body model, four sensors were placed on body segments (head, upper arm, forearm and hand). The system was calibrated before the kinematic testing protocol was implemented. To improve the measurement quality, the device automatically filtered raw data using Kalman filter algorithm.

Participants sat in a height-adjustable chair with their hips and knees flexed to 90°. Positions were not restrained, and compensatory movements were allowed when necessary (Li et al., 2015). Upper extremity maintained in the neutral position, with elbow extension and palm downward initially. The standardized procedure for the finger-to-nose test was introduced by the same researcher, and then was imitated by the participants for three times before the test. On a verbal command, the participants performed FNT as quickly and as accurately as possible, and then returned to the initial posture. Stroke individuals performed the test with the affected arm and the healthy adults performed with the non-dominant arm. The tests were recorded for five times, but a mean of three middle trials was used in statistical calculations (Alt Murphy et al., 2012; Schiefelbein et al., 2019).

### Kinematic Analysis

Kinematic analysis focused on UE end-point performance during the going phase of finger-to-nose test. Data recorded in the IMU software were exported to single.csv files, then were imported to and extracted through a semi-automated custom written program in MATLAB (The MathWorks, Natick, Massachusetts, United States) for kinematic analysis. Onset and end of movements were defined using a velocity threshold of 50 mm/s (Menegoni et al., 2009; Schiefelbein et al., 2019). UE kinematic metrics were calculated through the anatomical coordinate system and joint rotation recommended by the International Society Biomechanical (ISB) (Wu et al., 2005). In this study, six kinematic metrics were utilized: movement time (MT), mean velocity (VM), peak velocity (VP), percentage of time to peak velocity (TVP%), number of movement units (NMU) and normalized integrated jerk (NIJ) (Nordin et al., 2014).

MT is an objective and quantitative variable frequently used to reflect movement performance, which was defined as the time taken during the going phase of the test. To define VP, the maximum tangential velocity of the index finger was calculated during each movement segment; and VM was defined as the average tangential velocity. TVP% is the proportion of time spent during the start of the movement until the peak velocity. The number of velocity peaks characterize NMU over a cut-off value corresponding to the 10% of VP. When multiple velocity peaks occur, the movement is composed of several smaller, corrective sub movements. NIJ was utilized to assess movement smoothness, which was calculated using the jerk normalized by MT and length of the task (Johansson et al., 2017; Rodrigues et al., 2017),

$$\text{N}\,I\,J=\sqrt{\frac{\text{M}\,T^{5}}{2\times \text{l}\,e\,n\,g\,t\,h^{2}}\times \sum \text{j}\,e\,r\,k\,{\left(t\right)}^{2}}$$

where jerk is the third derivate of the endpoint displacement and length is the shortest distance between the start and end positions of the index finger.

### Statistical Analysis

Statistical analyses were performed on SPSS version 22.0 and R statistical software. A two-sided p-value of less than 0.05 was set as statistical significance. Categorical variables were compared through Chi squared test, and quantitative variables were compared through one-way ANOVA. The Shapiro-Wilk test was employed to evaluate the normal distribution of quantitative data. The Pearson’s correlation coefficients were conducted between the kinematic variables and clinical assessments. The limit for multicollinearity between independent variables was set at 0.7 for Correlation Coefficients.

Data were scaled into a matrix at first because the mean and variance may differ greatly across the variables. Data matrix was calculated using the PCA function of R software. Then the matrix underwent eigenvalue decomposition to obtain its eigenvectors with corresponding eigenvalues. Eigenvector represented the contribution of each kinematic variable to the principal component, and was visualized by the Correlation Circle. Eigenvalue represented the amount of variance explained by the PCs. The model utilized the least number of PCs to achieve ≥90% of the total variance explained. Finally, the original data set was transformed via the eigenvectors as weighting coefficients to obtain principal component scores (Kassambara, 2017). Wilcoxon rank-sum tests were conducted to detect subgroup differences in principal component loadings in age (<50 vs. ≥50), paretic side, type of stroke for stroke participants, and between the groups. Kruskal-Wallis tests were performed to assess the differences in stroke severity (FMA-UE scores 0–22, 23–47, 48–64). The obtained PCs were included as independent variables in multivariable regression to investigate the association between kinematic metrics and clinical assessments. Probability for entry in backward regression was set at 0.05 and removal at 0.10. Adjusted R^2^ values with *p*-values, unstandardized coefficient (β), and unique partial correlation coefficients were used to estimate the contribution of each PC to the model.

## Results

### Demographics and Clinical Characteristics

**FIGURE 1:**
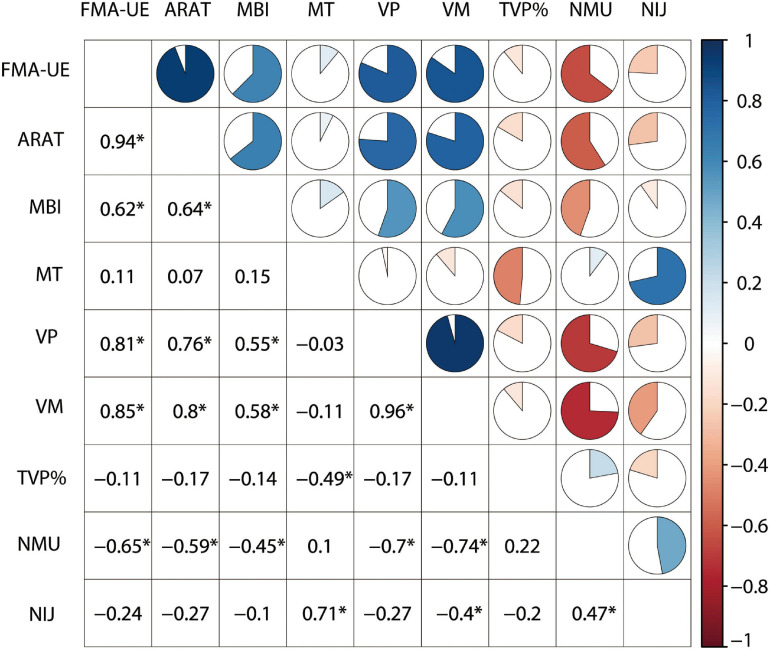
Correlations between clinical assessments and kinematic metrics.

Demographics and clinical characteristics of the participants were presented in Table 1. In this study, individuals with subacute stroke had a moderate UE impairment, with an average FMA scores of 36.22 ± 17.69 and ARAT scores of 23.97 ± 17.38. No statistical difference was observed in age, gender, Body Mass Index and TVP% between healthy participants and stroke individuals. The healthy participants performed the task with higher speed (VP, VM), less time (MT), and better smooth profiles (NMU and NIJ) than the stroke individuals (*P* < 0.001). Multicollinearity was found between MT and NIJ, as well as among VM, VP and NMU. Significant correlations were found between FMA-UE and VP (*r* = 0.81), VM (*r* = 0.85), and NMU (*r* = − 0.65). ARAT showed significant correlation with VP (*r* = 0.76), VM (*r* = 0.8), and NMU (*r* = − 0.59). MBI showed significant correlation with VP (*r* = 0.55), VM (*r* = 0.58), and NMU (*r* = − 0.45). MT, TVP% and NIJ were not significantly associated with the clinical assessments (Figure 1).

**TABLE 1:** Demographics and Clinical Characteristics.

Characteristics	Stroke group (*n* = 37)	Control group (*n* = 20)	*P*-value
Age (years)	49.78 ± 10.26	52.62 ± 10.23	0.318
Gender (M/F)	28/9	12/8	0.319
Body mass index (kg/m^2^)	24.43 ± 2.60	23.48 ± 2.64	0.195
MT (s)	1.09 ± 0.31	0.62 ± 0.12	*P* < 0.001*
VP(m/s)	1.61 ± 0.92	4.04 ± 0.67	*P* < 0.001*
VM (m/s)	0.78 ± 0.44	2.19 ± 0.39	*P* < 0.001*
TVP% (%)	42.23 ± 11.30	46.74 ± 5.16	0.156
NMU	2.56 ± 1.25	1.14 ± 0.28	*P* < 0.001*
NIJ	2.86 ± 1.98	0.54 ± 0.18	*P* < 0.001*
Days between onset and enrollment	106.30 ± 65.46	–	–
Type of stroke (ischemic/hemorrhagic)	26/11	–	–
Paretic side (left/right)	22/15	–	–
MMSE (range 0–30)	27.16 ± 2.41	–	–
FMA-UE (range 0–66)	36.22 ± 17.69	–	–
ARAT (range 0–57)	23.97 ± 17.38	–	–
MBI (range 0–100)	72.30 ± 22.20	–	–

Values are presented as means ± standard deviation or as otherwise indicated. MT, movement time; VP, peak velocity; VM, mean velocity; TVP%, percentage of time to peak velocity; NMU, number of movement units; NIJ, normalized integrated jerk; MMSE, Mini-Mental State Examination; FMA-UE, Fugl-Meyer Assessment for Upper Extremity; ARAT, Action Research Arm Test; MBI, Modified Barthel Index. *P < 0.05.

### Principal Component Analysis

**FIGURE 2:**
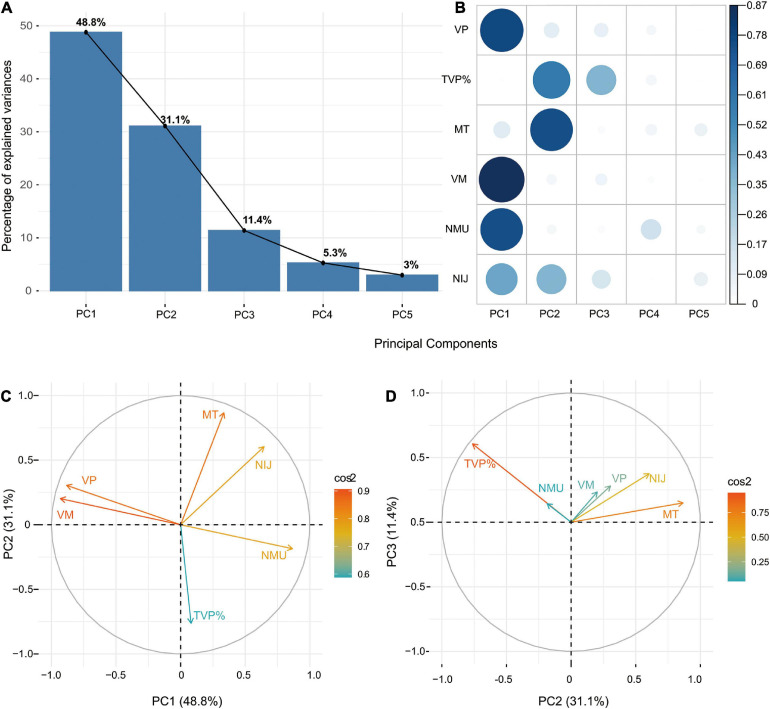
**(A)** Scree Plot demonstrating the principal components accounting for variances. **(B)** Squared coordinates demonstrating the proportion of representation of the kinematic variables to the PCs. **(C)** Correlation circles demonstrating the similarity in loading weights among correlated kinematic variables in PC1 and PC2. **(D)** Correlation circles demonstrating the similarity in loading weights among correlated kinematic variables in PC2 and PC3.

As shown in the Scree Plot (Figure 2A), based on eigenvalue decomposition of the kinematic metrics, the principal components for stroke participants were arranged in the descending order. The first three PCs explained 91.3% variance of the dataset. The quality or proportion of representation of the kinematic variables to the PCs were presented in the squared coordinates (Figure 2B). PC1 accounting for 48.8% of the variance was characterized by velocity profiles (VM, VP) and smoothness profiles (NMU, NIJ). PC2 accounting for 31.1% of the variance reflected movement planning (TVP%) and movement time (MT) of stroke survivors. PC3 accounting for 11.4% of the variance mainly described movement planning (TVP%).

The z-scores of each kinematic metric in accounting for the variance of the principal components for the stroke group and control group were demonstrated in the principal component loadings (Supplementary Figures 1–7 and Supplementary Tables 1, 2). No subgroup differences were found in principal component loadings concerning age, paretic side and type of stroke for stroke participants (Supplementary Figures 8–10). Stroke severity measured by the FMA-UE was found to be associated with the principal component loadings (Supplementary Figure 11 and Supplementary Tables 3–5). Besides, correlation circles demonstrated the similarity in loading weights among correlated kinematic variables in the respective PCs (Figures 2C,D). Positively correlated kinematic variables were grouped together and negatively correlated variables were positioned on opposite quadrants of the plot. PC1 was positively associated with the velocity variables (VM, VP) and negatively associated with the smoothness variables (NMU, NIJ). PC2 was positively associated with the TVP% and negatively associated with the MT. PC3 was positively associated with all the kinematic variables.

### Association With Clinical Assessments

The first three PCs were included in the multivariable regression models with clinical assessments as the dependent variables, including the FMA-UE, ARAT, and MBI. The results and equations of principal component regressions were presented in Table 2 (*P* < 0.001). PC1 was positively correlated with the clinical assessments and PC2 was negatively correlated. PC3 was positively correlated with the FMA-UE. The backward multiple regression indicated that principal components could explain the most variance in the assessment of motor impairment measured by the FMA-UE. The principal components together explained 71% of the total variance, which demonstrated a unique contribution of 55, 9, and 7%, respectively. In the model of ARAT, the PC1 and PC2 showed significant contribution to the model and explained 59% of the variance, accounting for 51 and 8%, respectively. In the model of MBI, PC1 and PC2 showed significant contribution to the model and explained 29% of the variance, accounting for 22 and 7%, respectively.

**TABLE 2:** Multivariable regression analysis of the principle components against the clinical assessments.

Independent	Unstandardized	Standard	Partial unique	*P*-value of	Adjusted R^2^
variables	coefficient β	error	contributions	the variable	(model *P-*value)
**Dependent variable: z-score of FMA-UE**					0.71 (<0.001*)
PC1	0.44	0.05	55%	< 0.001*	
PC2	–0.23	0.07	9%	0.002*	
PC3	0.34	0.11	7%	0.004*	
**Equation via inverse transformation:**					
FMA-UE = 7.24VP + 16.14TVP% + 6.79MT + 14.69VM − 2.74NMU + 0.79NIJ + 3.50					
**Dependent variable: z-score of ARTA**					0.59 (<0.001*)
PC1	0.42	0.06	51%	< 0.001*	
PC2	–0.21	0.08	8%	0.012*	
**Equation via inverse transformation:**					
ARAT = 4.76VP - 20.87TVP% + 2.86MT + 10.11VM − 3.33NMU − 0.57NIJ + 23.75					
**Dependent variable: z-score** of MBI					0.29 (0.001*)
PC1	0.29	0.08	22%	0.001*	
PC2	–0.22	0.10	7%	0.044*	
**Equation via inverse transformation:**					
MBI = 4.54VP − 26.21TVP% + 5.80MT + 9.39VM − 3.09NMU − 0.14NIJ + 70.62					

*FMA-UE, Fugl-Meyer Assessment for Upper Extremity; ARAT, Action Research Arm Test; MBI, Modified Barthel Index; PC, principal component; MT, movement time; VP, peak velocity; VM, mean velocity; TVP%, percentage of time to peak velocity; NMU, number of movement units; NIJ, normalized integrated jerk. ^∗^P < 0.05.*

## Discussion

Conventional multivariable analyses of kinematic data have to meet the criteria of statistical approaches. Potential meaningful variables may be excluded due to high-mathematical collinearity. In this study, the associations between six FNT kinematic variables and UE motor function were explored through PCA for individuals after subacute stroke. Our results showed that the first three principal components explaining 91.3% variance were significantly associated with the clinical assessments for the stroke individuals. The variance explained by principal component regression in FMA-UE (*R*^2^ = 0.71) were higher than ARAT (*R*^2^ = 0.59) and MBI (*R*^2^ = 0.29).

### PC1—Movement Speed and Smoothness

PC1 accounting for 48.8% variance of the data, was largely dominated by variables that described the movement speed and smoothness. Speed indexes reflect one’s efficiency and ease of movement. Similarly, mean and peak speed have been reported to correlate with upper limb motor impairment in a previous study (Bosecker et al., 2010). Movement speed depends on individual’s voluntary effort, ability to control interaction torques of agonist/antagonist muscles and maintain normal inter-joint coordination during timed tasks (Nordin et al., 2014). Besides, smoothness is related to the temporal organization or coordination of upper-limb segments since post-stroke individuals typically present excessive discrete movements (Balasubramanian et al., 2015).

Correlation analysis showed that movement speed was negatively associated with NMU (*r* = −0.70 and −0.74) in stroke survivors. However, the correlation analysis was inconsistent with the final equations shown in Table 2 because there may be intrinsic interactions among variables. A possible explanation may be the case that movement smoothness was sacrificed for increased speed in some participants (Swaine and Sullivan, 1993). However, lower speed cannot ensure increased performance in smoothness as measured by NIJ. It should be noted that NIJ showed not significant association with peak velocity. In addition, measurement of smoothness should be taken with caution because a single smoothness parameter cannot reflect the entire recovery process of stroke survivors (Rohrer et al., 2002). Therefore, smoothness and speed indexes should be in combination as a major kinematic component (PC1) to depict only part of UE performance.

### PC2 and PC3—Movement Planning and Time

PC2 accounting for 31.1% variance, was largely dominated by movement planning and movement time; PC3 accounting for 11.4% of the variance mainly described movement planning. TVP% reflects movement planning and is defined as the proportion of time spent from the onset to the peak velocity (Nordin et al., 2014). MT refers to temporal efficiency to perform a certain activity or movement, and is expected to decrease with patient’s recovery (Zollo et al., 2011). Compared with healthy adults, post-stroke individuals had prolonged movement duration while the left-shifted velocity peaks were not statistically significant. In the current study, the clinical scales showed weak correlations with TVP% and MT, which were not included in the conventional regression models. However, PC2 and PC3 increased the performance of regression models by 7–9%. This is consistent with a study of robot-based kinematic assessment that movement duration can add value to estimate FMA-UE (Bosecker et al., 2010). The results therefore indicated that PC2 and PC3 may contain a considerable proportion of kinematic information, which should be taken into account when interpreting and estimating UE motor function.

In line with our study, these kinematic variables, especially velocity profiles, have been previously reported to affect UE motor function after stroke (van Dokkum et al., 2014; Schwarz et al., 2019). However, the associations between kinematics and some clinical scales are often weak to moderate and even controversial (Tran et al., 2018), e.g., NIJ and jerk (Rohrer et al., 2002; Gulde and Hermsdörfer, 2018), NMU (Rohrer et al., 2002; Otaka et al., 2015), and VP (Gilliaux et al., 2014). Collinearity among these variables, like VM, VP, and NMU, makes it difficult for conventional multivariate statistical models to explain heterogeneous population. Our results indicated that the UE motor function may be associated with multiple variables contained in the kinematic patterns named principal components, instead of separate parameters. In addition, multiple kinematic metrics were weighted and considered as part of the PCs to estimate clinical scales. The same kinematic variables contributed differently to each principal components, suggesting that the intrinsic correlations among variables could exert influence on UE motor function. Equations acquired from PCA-based regression are important for understanding UE motor control during FNT that is often ignored in conventional statistical models. Moreover, the MBI is a questionnaire for ADL instead of an observational measure toward UE motor function. Hence, individuals could have used compensatory behaviors or actually the less affected arm to improve the score, which may be hardly illustrated by the present kinematic assessment (Hsieh et al., 2007).

Our results showed that FNT kinematics could explain more variances in aspects of motor impairment as measured by FMA-UE, than activity assessments as measured by ARAT and MBI. According to our best knowledge, there was no previous report on principal component regression for end-point kinematics of gross movement obtained in subacute stroke survivors. In studies using multivariable linear regression, various task settings were implemented to investigate the variances of clinical scores explained by kinematics. Similarly, the FMA-UE was well explained by trunk displacement and shoulder flexion (51%) for the pointing task, and by trunk displacement alone (52%) for the reach-to-grasp task (Subramanian et al., 2010). In a drinking task, movement smoothness and trunk displacement together explain 67% of the total variance in functional assessment (ARAT), while trunk displacement alone explained 20% of the variance in motor impairment (FMA-UE) (Alt Murphy et al., 2012). The associations between kinematic variables and the capacity activity were relatively low in our study, suggesting that the kinematic testing protocols may be task-specific to measure different aspects of ICF domains after stroke. In addition, FMA-UE and ARAT were only explained 20 and 13% of the variance in a manual dexterity task with relatively small workspace (Hussain et al., 2019). The varying correlations between kinematics and clinical scales indicate that kinematic tests may likewise measure different ICF domains, which should be taken into consideration the task selection and clinical interpretation of kinematic analysis.

## Limitations

One of the limitations of this study is the relatively limited sample size. Although no subgroup differences were found in principal component loadings concerning age, paretic side and type of stroke for stroke participants, the results must be interpreted with caution when generalizing to a wider range of populations. Moreover, there are currently no guidelines for selecting standardized kinematic assessments and the optimal kinematic metrics. Our results are limited to the similar end-point movement performance of kinematic test and comparable variables utilized during the FNT. Future studies should therefore include much variables (such as the limit of arm movement), comprehensive tasks at different UE segments as well as trunk movement and ICF levels (WHO, 2001; Tran et al., 2018).

## Conclusion

This study showed that kinematic components during finger-to-nose test identified through PCA are associated with upper extremity motor function. PCA-based regression model indicates that finger-to-nose kinematics reflecting movement strategy, smoothness and velocity, measure much aspects of motor impairment than activity assessments. Such machine-learning method reveals the intrinsic association among kinematic metrics including velocity, smoothness and movement strategy. Our findings provide a new perspective on UE clinical assessment and future rehabilitation targeted for principal components of kinematic metrics.

## Data Availability Statement

The data files are available from the corresponding author upon reasonable request.

## Ethics Statement

The studies involving human participants were reviewed and approved by the Ethical Committee of the Tongji Medical College, Huazhong University of Science and Technology. The patients/participants provided their written informed consent to participate in this study.

## Author Contributions

ZJC, CH, JX, and XL-H conceived the study design. ZJC, CH, NX, MHG, and YAL performed the clinical trials. ZJC and CH analyzed the results, and involved in the interpretation of the results. CH and CHX provided the technical advice for the analyses. ZJC, JX, and XLH drafted the manuscript. All authors contributed to manuscript revision, read, and approved the submission of the manuscript.

## Conflict of Interest

The authors declare that the research was conducted in the absence of any commercial or financial relationships that could be construed as a potential conflict of interest.
