# Supplementary material for: Association Between Finger-to-Nose Kinematics and Upper Extremity Motor Function in Subacute Stroke: A Principal Component Analysis
Source: Front Bioeng Biotechnol. 2021 Apr 12;9:660015. doi: 10.3389/fbioe.2021.660015 (PMC8072355; doi:10.3389/fbioe.2021.660015)
Supplement: Supplementary file 1 [file Data_Sheet_1.docx]

***Supplementary Material***

**1. Quality of representation of the variables on principal components for the Stroke Group.**


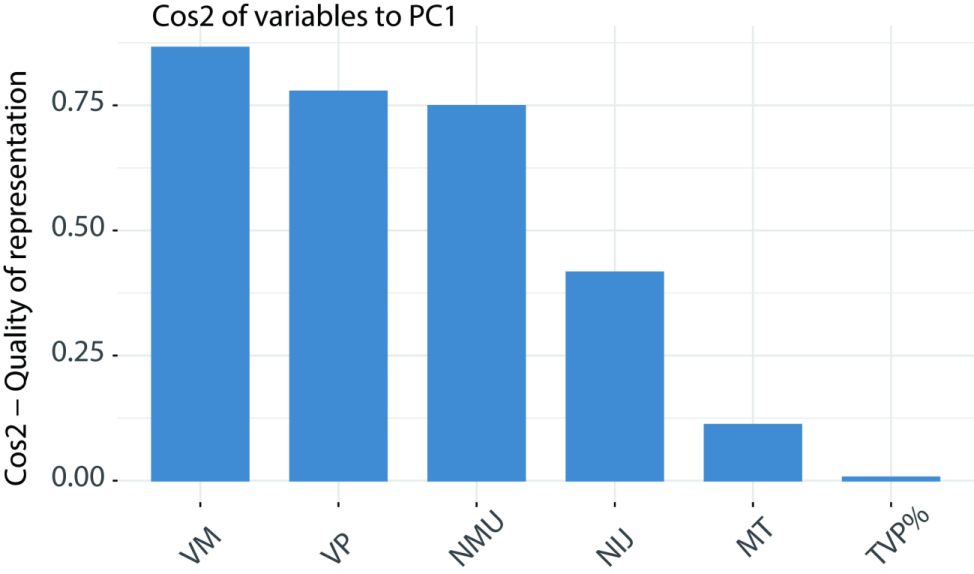


**Supplementary Figure 1.** Quality of representation of the variables on principal component 1.


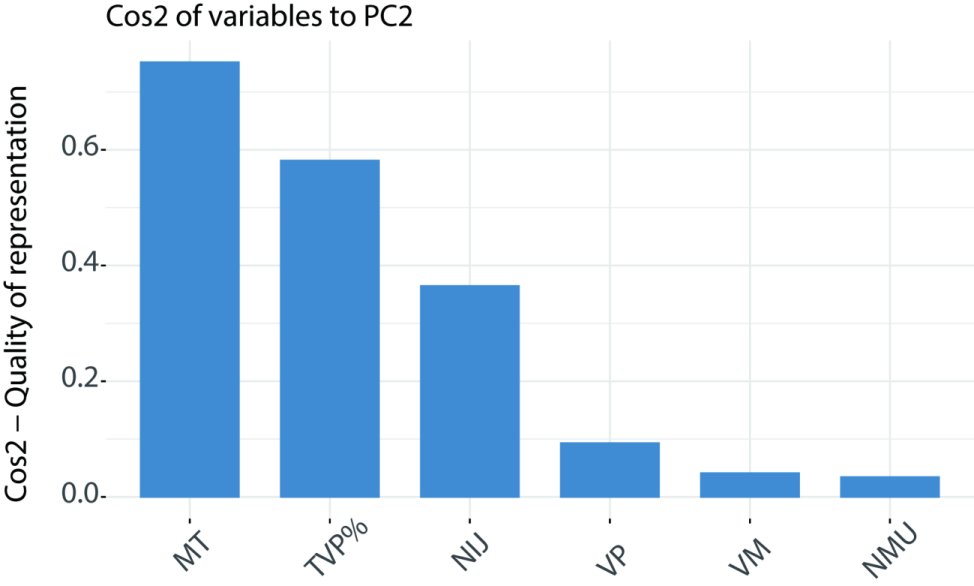


**Supplementary Figure 2.** Quality of representation of the variables on principal component 2.


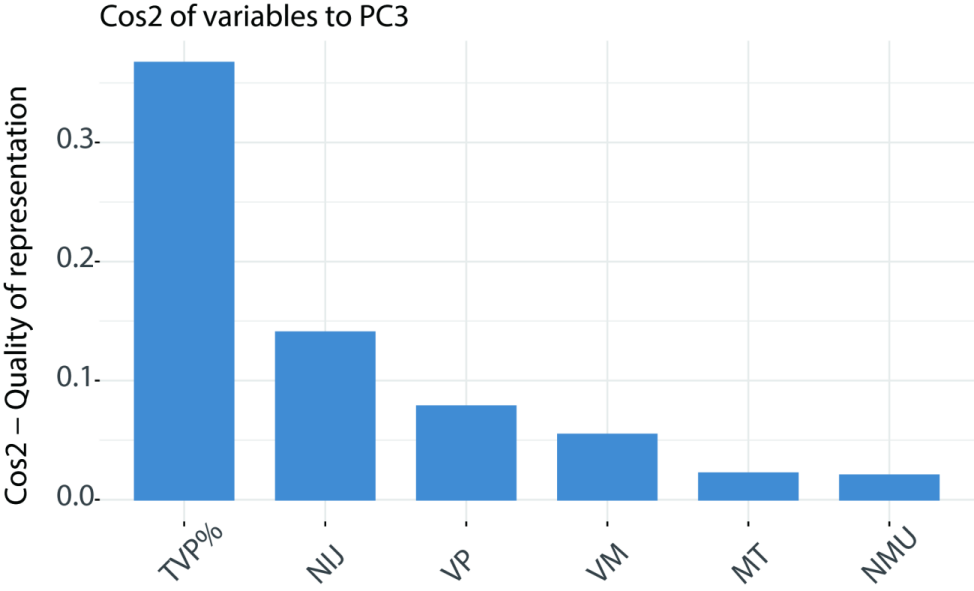


**Supplementary Figure 3.** Quality of representation of the variables on principal component 3.


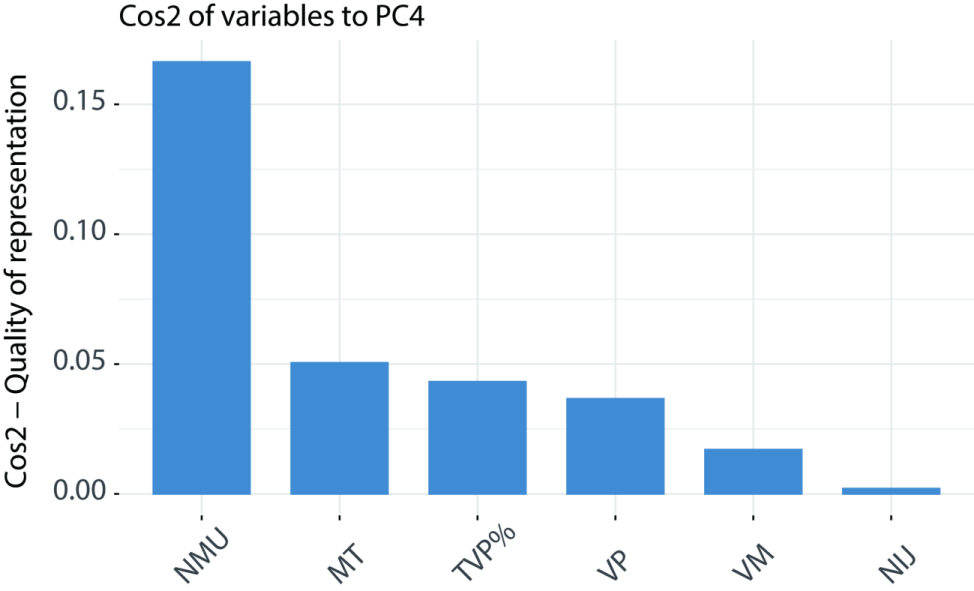


**Supplementary Figure 4.** Quality of representation of the variables on principal component 4.


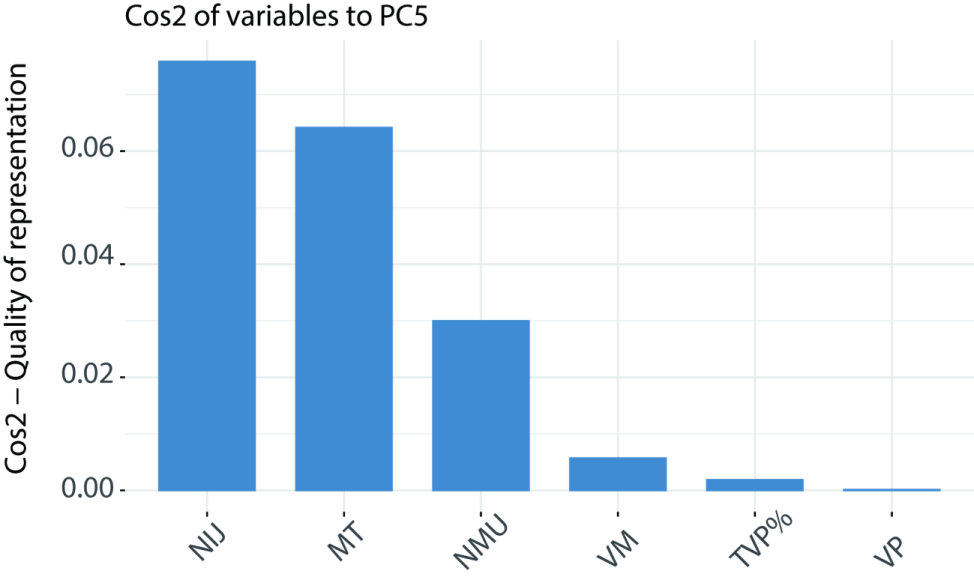


**Supplementary Figure 5.** Quality of representation of the variables on principal component 5.


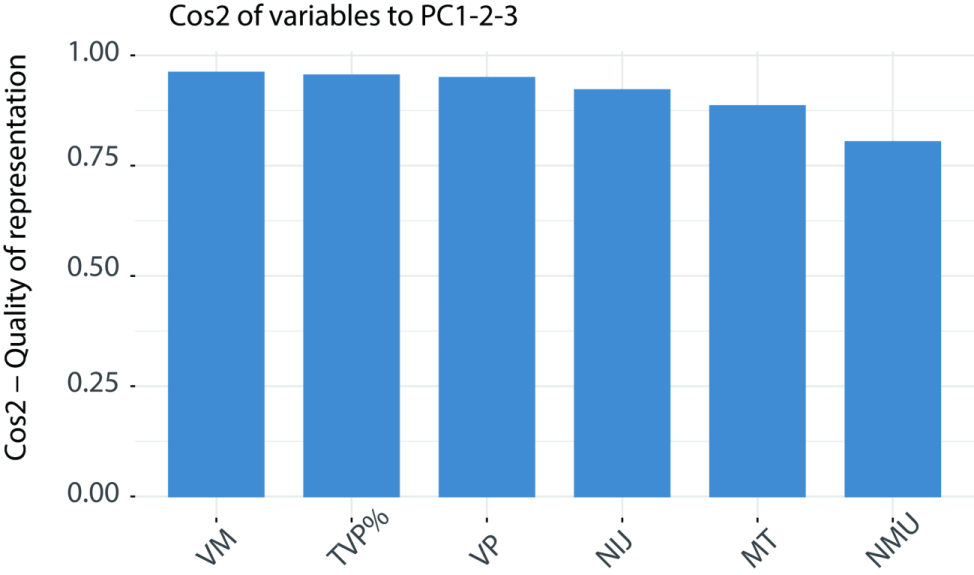


**Supplementary Figure 6.** Quality of representation of the variables on each principal component1-2-3.

**2. The comparison of PCA for the Stroke group and Control group.**


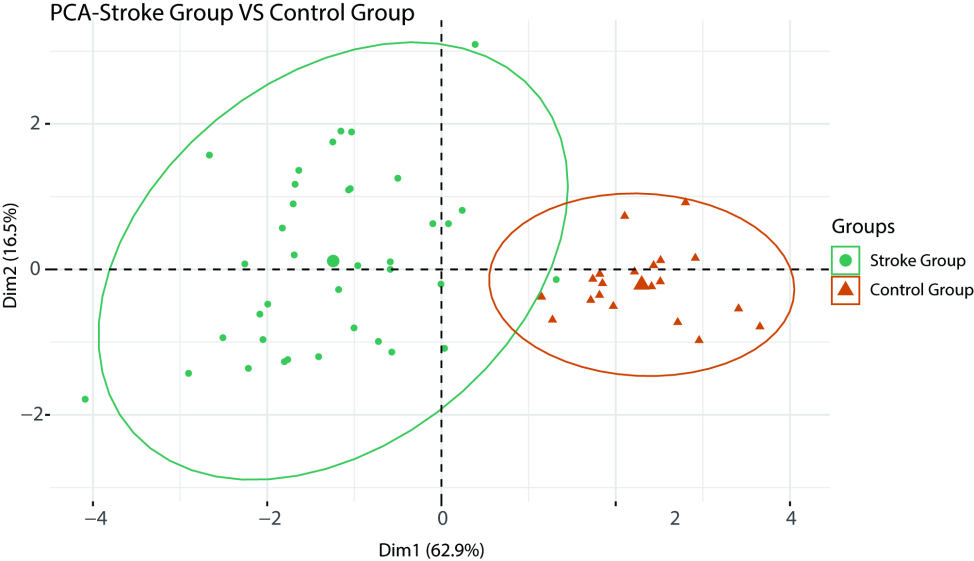


**Supplementary Figure 7.** PCA- Stroke Group VS Control Group.

**Supplementary Table 1.** Principal Component Loadings Demonstrating the Weighting Coefficient of Each Kinematic Metric (z-score) to Obtain the Principal Components for the Stroke Group.

| Characteristics | PC1* | PC2* | PC3* | PC4 | PC5 |
| --- | --- | --- | --- | --- | --- |
| VP (mm/s) | 0.52 | -0.22 | 0.34 | -0.34 | 0.03 |
| TVP% (%) | -0.05 | 0.56 | 0.73 | 0.37 | -0.10 |
| MT (s) | -0.20 | -0.63 | 0.18 | 0.40 | -0.60 |
| VM (mm/s) | 0.54 | -0.15 | 0.28 | -0.23 | -0.18 |
| NMU | -0.51 | 0.14 | 0.17 | -0.73 | -0.41 |
| NIJ | -0.38 | -0.44 | 0.45 | -0.08 | 0.65 |

Abbreviations: MT: movement time; VP: peak velocity; VM: mean velocity; TVP%: percentage of time to peak velocity; NMU: number of movement units; NIJ: normalized integrated jerk. *: Principal components achieving ≥ 90% of the total variance explained.

**Supplementary Table 2.** Principal Component Loadings Demonstrating the Weighting Coefficient of Each Kinematic Metric (z-score) to Obtain the Principal Components for the Control Group.

| Characteristics | PC1* | PC2* | PC3* | PC4* | PC5 |
| --- | --- | --- | --- | --- | --- |
| VP (mm/s) | 0.52 | 0.42 | 0.23 | -0.09 | -0.14 |
| TVP% (%) | 0.02 | 0.31 | 1.10 | -0.28 | 0.17 |
| MT (s) | 0.36 | -0.33 | -0.17 | 1.48 | 0.47 |
| VM (mm/s) | 0.82 | -0.23 | -0.16 | 0.62 | 1.46 |
| NMU | 0.11 | 1.17 | 0.18 | -0.28 | -1.23 |
| NIJ | 0.11 | 0.15 | 0.17 | -0.16 | 2.14 |

Abbreviations: MT: movement time; VP: peak velocity; VM: mean velocity; TVP%: percentage of time to peak velocity; NMU: number of movement units; NIJ: normalized integrated jerk. *: Principal components achieving ≥ 90% of the total variance explained.

**3. Subgroup analysis of PCA for stroke individuals.**

**3.1 Age.**


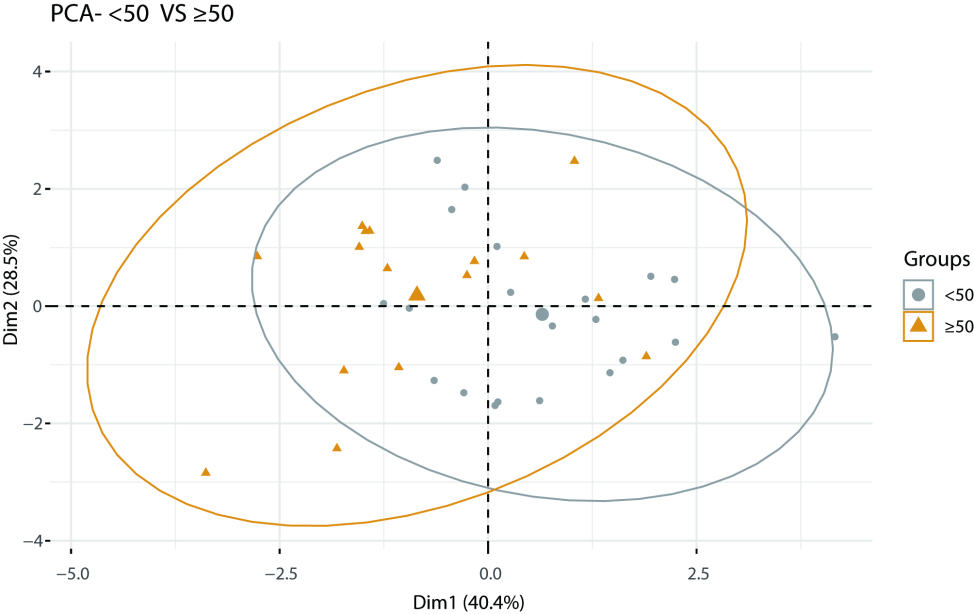


**Supplementary Figure 8. PCA- Age <50 VS** ≥**50.**

**3.2 Affected Side.**


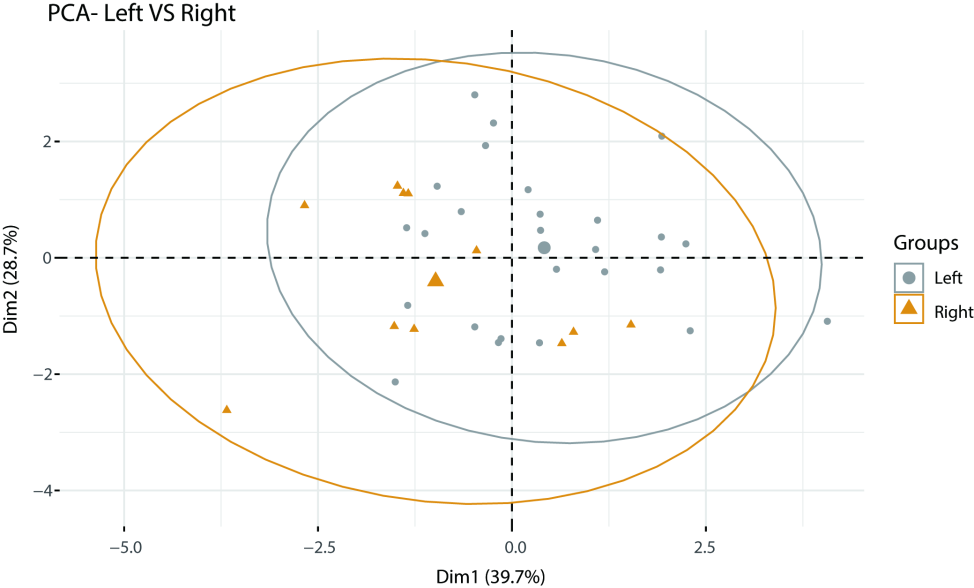


**Supplementary Figure 9. PCA- Left VS Right.**

**3.3 Stroke Type.**


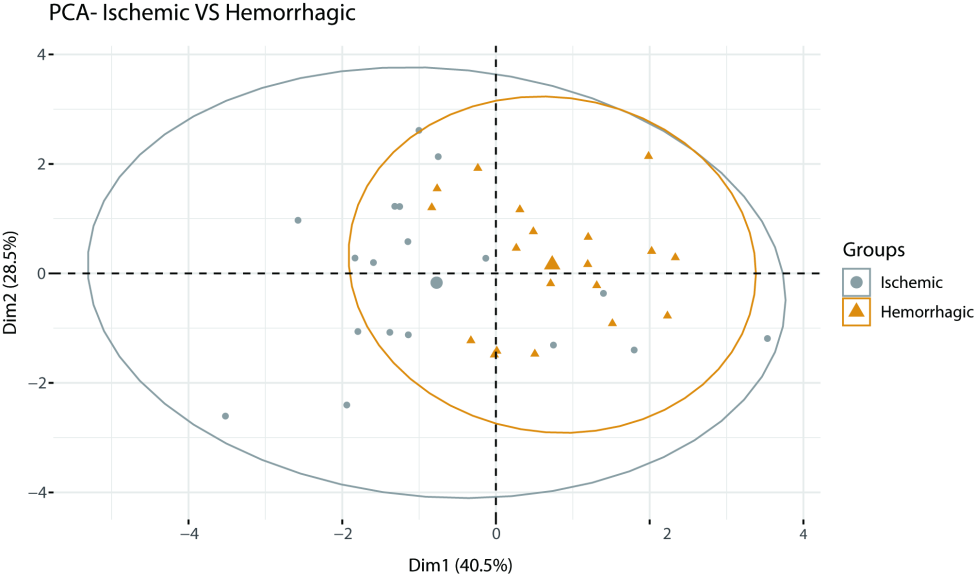


**Supplementary Figure 10. PCA- Ischemic VS Hemorrhagic.**

**3.4 Stroke Severity.**


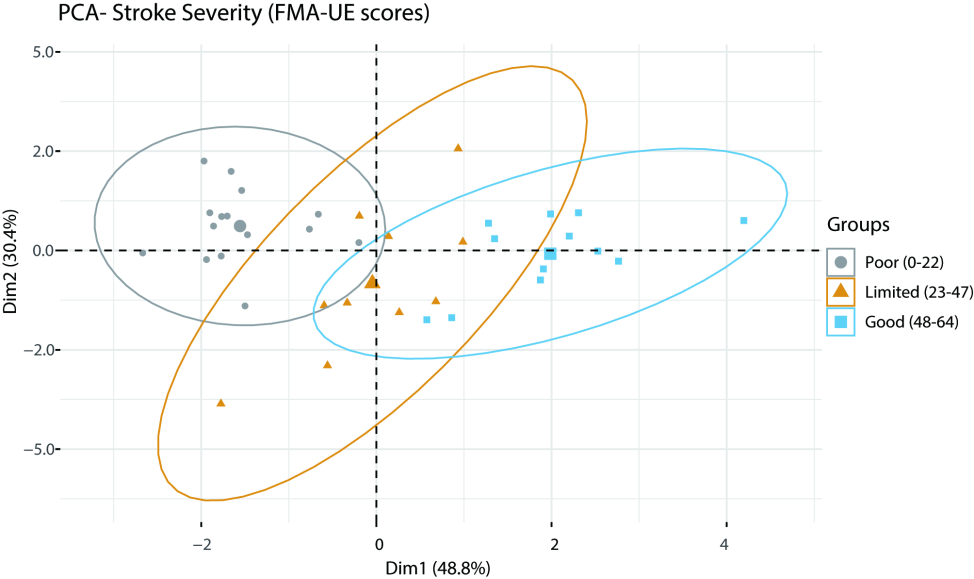


**Supplementary Figure 11. PCA- Stroke Severity.**

**Supplementary Table 3.** Principal Component Loadings Demonstrating the Weighting Coefficient of Each Kinematic Metric (z-score) to Obtain the Principal Components for the Stroke Participants with Poor FMA-UE scores (0-22).

| Characteristics | PC1* | PC2* | PC3* | PC4 | PC5 |
| --- | --- | --- | --- | --- | --- |
| VP (mm/s) | 0.47 | -0.09 | 0.46 | -0.30 | -0.10 |
| TVP% (%) | -0.42 | -0.09 | -0.22 | -0.84 | 0.22 |
| MT (s) | 0.18 | -0.63 | -0.03 | 0.08 | 0.08 |
| VM (mm/s) | 0.48 | 0.06 | 0.30 | -0.40 | 0.03 |
| NMU | -0.44 | -0.23 | 0.42 | -0.07 | -0.74 |
| NIJ | -0.21 | -0.59 | 0.32 | 0.15 | 0.48 |

Abbreviations: MT: movement time; VP: peak velocity; VM: mean velocity; TVP%: percentage of time to peak velocity; NMU: number of movement units; NIJ: normalized integrated jerk. *: Principal components achieving ≥ 90% of the total variance explained.

**Supplementary Table 4.** Principal Component Loadings Demonstrating the Weighting Coefficient of Each Kinematic Metric (z-score) to Obtain the Principal Components for the Stroke Participants with Limited FMA-UE scores (23-47).

| Characteristics | PC1* | PC2* | PC3* | PC4 | PC5 |
| --- | --- | --- | --- | --- | --- |
| VP (mm/s) | 0.24 | -0.22 | 0.75 | -0.14 | 0.15 |
| TVP% (%) | 0.32 | 0.51 | -0.18 | -0.63 | 0.45 |
| MT (s) | -0.48 | -0.16 | 0.18 | 0.10 | 0.68 |
| VM (mm/s) | 0.49 | -0.08 | 0.33 | -0.06 | -0.24 |
| NMU | -0.42 | 0.42 | 0.14 | -0.16 | -0.46 |
| NIJ | -0.43 | 0.07 | 0.37 | -0.46 | -0.15 |

Abbreviations: MT: movement time; VP: peak velocity; VM: mean velocity; TVP%: percentage of time to peak velocity; NMU: number of movement units; NIJ: normalized integrated jerk. *: Principal components achieving ≥ 90% of the total variance explained.

**Supplementary Table 5.** Principal Component Loadings Demonstrating the Weighting Coefficient of Each Kinematic Metric (z-score) to Obtain the Principal Components for the Stroke Participants with Good FMA-UE scores (48-64).

| Characteristics | PC1* | PC2* | PC3* | PC4 | PC5 |
| --- | --- | --- | --- | --- | --- |
| VP (mm/s) | 0.37 | 0.18 | -0.69 | 0.19 | -0.17 |
| TVP% (%) | 0.04 | -0.82 | 0.11 | -0.09 | -0.39 |
| MT (s) | -0.42 | 0.21 | -0.11 | -0.62 | 0.20 |
| VM (mm/s) | 0.43 | -0.21 | -0.40 | -0.24 | 0.26 |
| NMU | -0.36 | -0.45 | -0.39 | 0.02 | 0.53 |
| NIJ | -0.44 | 0.06 | -0.42 | -0.17 | -0.65 |

Abbreviations: MT: movement time; VP: peak velocity; VM: mean velocity; TVP%: percentage of time to peak velocity; NMU: number of movement units; NIJ: normalized integrated jerk. *: Principal components achieving ≥ 90% of the total variance explained.
